# NK cells are negatively regulated by sCD83 in experimental autoimmune uveitis

**DOI:** 10.1038/s41598-017-13412-1

**Published:** 2017-10-16

**Authors:** Wei Lin, Xuejing Man, Peng Li, Nannan Song, Yingying Yue, Bingqing Li, Yuanbin Li, Yufei Sun, Qiang Fu

**Affiliations:** 1 Department of microbiology, Institute of Basic medicine, Shandong Academy of medical Sciences, Jinan, 250032 China; 2 Department of Ophthalmology, Yuhuangding Hospital, Yantai, 264001 China; 3 Department of Immunology, Binzhou Medical University, Yantai, 264003 China

## Abstract

Natural killer (NK) cells represent a subset of lymphocytes that contribute to innate immunity and have been reported to play a role in autoimmune uveitis. However, the mechanisms regulating NK cellular function in this condition remain unclear. Herein, we investigated the status of NK cells in experimental autoimmune uveitis (EAU). We found that the number of CD83^+^CD3^−^NK1.1^+^ cells was increased in the inflamed eyes and spleens of the EAU mouse model. At the recovery stage of EAU, serum concentrations of soluble CD83 (sCD83) were increased. sCD83 treatment relieved retinal tissue damage and decreased the number of infiltrating NK cells in inflamed eyes. Further analysis of the effects of sCD83 treatment in EAU revealed that it reduced: 1) the expressions of CD11b and CD83 in NK cells, 2) the percent of CD11b^high^CD27^low^CD3^−^NK1.1^+^ cells and 3) the secretion of granzyme B, perforin and IFN-γ in NK cells as demonstrated both *in vivo* and *in vitro*. When sCD83 treated-NK cells were transferred into EAU mice, retinal tissue damage was relieved. These results demonstrate sCD83 down-regulate NK cellular function and thus provide important, new information regarding the means for the beneficial effects of this agent in the treatment of autoimmune uveitis.

## Introduction

Nature killer (NK) cells are a part of the innate immune system and represent the first line of defense against infections. The major role of NK cells involves a cytotoxic reaction against neoplastic, infected or auto-reactive cells. Recently, NK cells have also been implicated in the regulatory mechanisms of autoimmune diseases such as diabetes and insulitis, rheumatoid arthritis, experimental autoimmune encephalomyelitis (EAE) and experimental autoimmune uveitis (EAU)^1–5^. Findings from these studies have resulted in the hypothesis that NK cells may change the balance of immunity in autoimmune diseases by regulating the secretion of cytokines or by interacting with other cells^1,4,6–10^. Accordingly, regulation of NK cellular function could prove beneficial in contributing to the treatment of autoimmune diseases. However, the mechanisms involved with regulating NK cellular function remain unclear.

Many factors are involved in mediating the function of NK cells. For example, the expression of surface molecules on NK cells, including activating signals, inhibitory signals, mature makers and adhesion molecules, can all act as important factors in determining NK cellular functions^11^. CD69, C type lectin receptors (NKG2D, CD94-NKG2C), IgG-like receptors (2B4), and Killer cell immunolglobulin-like receptors (KIR) are known to be activating signals which can play an important role in NK cell activation, while inhibitory receptors present on NK cells including C-type lectin receptors (CD94-NKG2A), cytotoxicity receptors (NKp64, NKp44) and the major histocompatibility complex class I (MHC I)^12–14^. Ultimately, the balance between activating and inhibitory signals on NK cells determines the cytotoxic reaction of NK cells against neoplastic, infected or auto-reactive cells^11^. Additional factors requiring consideration include, CD27 (a member of the tumor necrosis factor receptor super-family) and CD11b, which are mature markers of NK cells. The surface densities of CD27 and CD11b on NK cells represent the level of maturation and function of NK cells, and divides NK cells into four subsets^15–18^. These four subsets are correlated with different functions of NK cells. CD11b^high^CD27^low^ NK cells represent a mature subset which exerts a strong effect upon cytotoxic reactions and cytokine secretion^17^. CD11b^high^CD27^high^ NK cells and CD11^low^CD27^high^ NK cells produce interferon gamma (IFN-γ)^17,19^. CD11b^low^CD27^low^ NK cells display an immature phenotype and possess the potential for differentiation^17^. Factors which can regulate the maturation of NK cells and/or the proportion of NK-cell subsets may thus have the potential of altering NK cell function.

Recently, the soluble CD83 (sCD83) molecule has been demonstrated to exert strong anti-inflammatory reactions and can act as an immuno-suppressive mediator to regulate the activation and maturation of dendritic cells (DCs) and monocytes^20,21^. Moreover, it has been reported to be a potential therapeutic agent for use in the treatment of autoimmune diseases^21–25^. sCD83 is a soluble molecule that is expressed in the extracellular domain of the membrane-bound CD83 (mCD83) molecule. mCD83 is a member of the immunoglobulin super family which is expressed on many activated cells, such as mature DCs, activated T cells and B cells, and activated NK cells^21–23,26,27^. Upon activation of these cells, mCD83 is found to be highly expressed on their surfaces. In turn, mCD83 is hydrolyzed by enzymes to product sCD83, which is secreted to regulate the maturation of NK cells. Elevated sCD83 binds with myeloid differentiation factor-2 (MD-2) to alter the TLR-4 signaling pathway to regulate the function of CD14+ monocytes^28^ and reduces expression levels of CD1a, CD80, CD86 and MHC II to inhibit monocyte differentiation into DCs^20^. sCD83 can also induce tolerogenic DCs and reduce the expressions of CD40, CD80 and CD83 on DCs, and, in this way, alter the maturation of DCs^21,22,27^. As activated NK cells can express CD83 on their surface^26^, it then seems likely that activated NK cells might produce sCD83 to regulate the status of NK cells. However, the mechanisms of these effects of sCD83 on NK cells remain unclear.

Autoimmune uveitis represents one of the autoimmune diseases which currently lacks a specific treatment^29,30^. In experimental autoimmune uveitis (EAU), NK cells are increased and aggravate the severity of EAU^31,32^. Findings from recent studies have indicated that a significant increase in activated NK cells was associated with the production of IFN-γ, which was correlated with the pathogenesis of Behcet’s disease, a type of autoimmune uveitis^5,33^. However, NK cells which failed to produce IFN-γ, were associated with a remission of autoimmune uveitis^5^. Alterations in the status of NK cells might also contribute to the recovery of Behçet’s disease through suppression of a Th1 response^5,33^. Whether sCD83 could affect the development of EAU through alterations in NK cell status is not known. Hence, in our study we examined the status of NK cells in EAU, a well-established rodent model for human autoimmune uveitis^34^. In addition, we investigated the effects of sCD83 on EAU and the status of NK cells. Our findings showed that sCD83 decreased the maturation and function of NK cells by decreasing the expression of the adhesion molecule, CD11b, on NK cells. Accordingly, the results from our studies support the hypothesis that sCD83 might be a potential therapeutic agent for use in the treatment of EAU.

## Results

### CD83^+^ NK1.1^+^ cells are increased in the eyes and spleen of the EAU model

**Figure 1:**
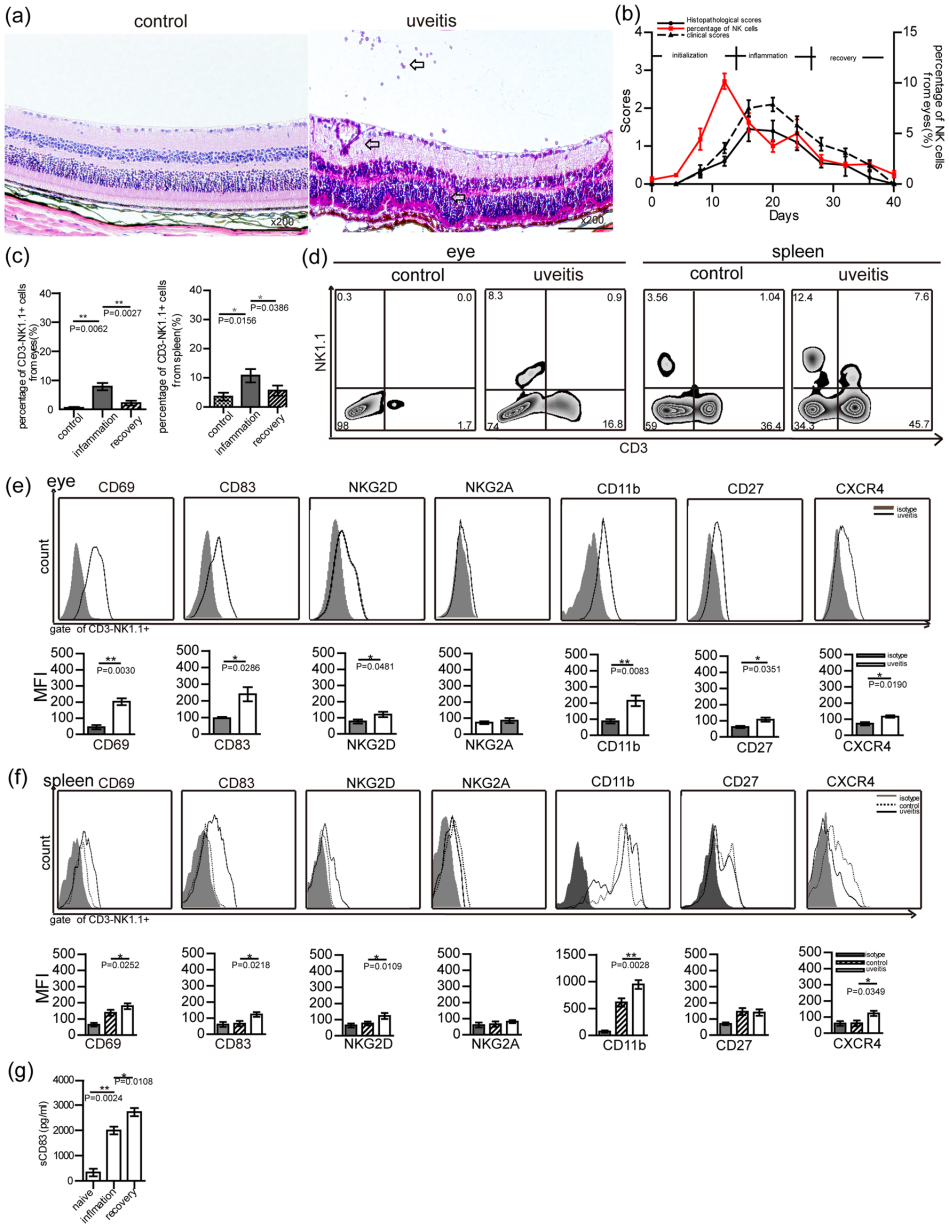
CD83^+^ NK cell numbers were increased in inflamed eyes and spleen cells within the EAU model. (**a**) Histopathology of a representative eye section from control (Day 0) and immunized mice on day 16 (hematoxylin and eosin, original magnification, ×200). Infiltrating lymphocytes, vasculitis and photoreceptor folding within the retina were found in the inflamed eye (hollow arrows). (**b**) Histopathological scores were evaluated in the development of uveitis disease (Three mice were used at every time point, and three separate experiments were repeated, as indicated by the black line), while the percent of CD3^−^NK1.1^+^ cells was determined within infiltrating cells in the eyes (red line). (**c**) The percent of CD3^−^NK1.1^+^ cells in total number of infiltrating eye or spleen cells of immunized mice as detected at the inflammatory (day 12–16) or recovery (day 28) stages, and compared with those in untreated naive mice (A total of ten mice/group were used and experiments were replicated three times, values represent the mean ± s.e.m., *P < 0.05, **P < 0.01). (**d**) The percent of CD3^−^NK1.1^+^ cells in inflamed eyes or spleen from a representative EAU and control mouse. (**e**) Expressions of CD69, CD83, NKG2D, NKG2A, CD11b, CD27 and CXCR4 in infiltrating CD3^−^NK1.1^+^ cells within the eye (a representative result from three experiments). Analysis of the mean fluorescence value (MFI) of these molecules on CD3^−^NK1.1^+^ cells (bottom row of Fig. 1e, a total of ten mice/group were used and experiments were replicated three times; mean ± s.e.m., *P < 0.05, **P < 0.01). (**f**) Expressions of CD69, CD83, NKG2D, NKG2A, CD11b, CD27 and CXCR4 on CD3^−^NK1.1^+^ cells in inflamed spleen as compared to that from control mice (a representative result from three experiments) and the mean fluorescence value (MFI) of these molecule (A total of ten mice/group were used and experiments were replicated three times, mean ± s.e.m. *P < 0.05, **P < 0.01). (**g**) Concentrations of sCD83 in the blood of EAU mice at the inflammatory and recovery stages were measured and compared with that in the blood of naive mice (A total of ten mice/group were used for three separate experiments, mean ± s.e.m., *P < 0.05, **P < 0.01).

The EAU model was induced by immunizing the peptides IRBP_1-20_ and PTX in C57BL/6 mice as previously described^35,36^. Inflamed eyes were harvested every four days after immunization and histopathological changes were analyzed. Compared with that of the normal eye, a large number of infiltrating lymphocytes were found within the posterior chamber and a disorganization of retinal tissue was observed in the inflamed eye (Fig. 1a, hollow arrows). Histological scores of retinal tissue from inflamed eyes rose by days 8–12 post-immunization (the initialization stage), peaked on days 16–24 (the inflammation stage) and dropped on day 28 (the recovery stage). Thus, maximal levels of inflammation were observed at 16–24 days post-immunization in this EAU model (Fig. 1b). At same time, clinical scores were also obtained to be shown in Fig. 1b.

In addition, the number of infiltrating cells in inflamed eyes increased rapidly during the initialization stage (days 8–12) as shown in Supplementary Fig. S1. A large number of lymphocytes were located within these inflamed eyes including CD3^+^ T cells, CD11c^+^ MHC-II^+^ DCs, CD3^−^ NK1.1^+^ cells, CD11b^+^ F4/80^+^ ly6c^−^ macrophages, CD11b^+^ F4/80^−^ ly6c^+^ monocytes/neutrophils and CD11b^+^ F4/80^+^ ly6c^+^ cells neutrophil granulocytes (Supplementary Fig. S2a). Each of these lymphocytes was also found to be increased in the inflamed spleen (Supplementary Fig. S2b). These findings reveal the specific types of immune disorders that are present in the systemic and local immune environment of this EAU model.

As NK cells have been shown to aggravate the severity of EAU^5,31–33^, the status of NK cells was analyzed in this model. NK1.1^+^ cells were increased in inflamed eyes on days 8–16 post-immunization (the initialization stage), with initial peak values being observed on day 12 (Fig. 1b). These changes in NK1.1^+^ cells were found to occur at earlier time points than that of the most severe ocular pathological changes as detected with H&E staining. While these numbers of NK1.1^+^ cells were decreased on day 20 post-immunization, a second peak was observed on day 24 (Fig. 1b) The majority of these increased NK cells at the initialization stage were CD3^−^ NK1.1^+^ cells, accounting for 7.84 ± 2.16% (mean ± s.e.m.) of the total number of eye-infiltrating cells (Fig. 1c,d), while CD3^+^ NK1.1^+^ cells accounted only for 1.25 ± 0.67% (mean ± s.e.m.) of the total number of eye-infiltrating cells. The number and phenotype of NK cells in the spleen cells were also analyzed. The number of spleen CD3^−^ NK1.1^+^ cells were significantly increased in response to this inflammation as compared with that observed in the control spleen (Fig. 1c,d, P = 0.0158).

As an approach to verify the role of NK cells in this EAU model, NK1.1^+^ cells were isolated from the inflamed spleen on days 12–16 post-immunization with use of a micro-beads kit and transferred into naive mice. A histopathological examination was performed on day 8 after NK1.1^+^ cell transferred and the severity of disease was analyzed. The severity of retinal tissue damage within the eyes of the mice receiving these NK1.1^+^ cells is presented in Supplementary Fig. S3a. The percent of lymphocyte subsets including CD3^+^ T cells, CD11c^+^ MHC-II^+^ DCs, CD11b^+^ ly6c^−^ F4/80^+^ marcophages and CD3^−^ NK1.1^+^ NK cells from the eyes and spleen of naive mice were increased in response to NK cell transfer (Supplementary Fig. S3b–d). Thus, increases in NK1.1^+^ cell number during the initialization stage might be considered as a pathological factor for the development of EAU.

To further analyze the phenotype of these increases in NK cells, we harvested the lymphocytes from inflamed eyes and spleen and measured the expressions of CD11b, CD27, CD69, CD83, NKG2D, and CXCR4 on CD3^−^ NK1.1^+^ cells with use of flow cytometry. Our results showed that the activating signals, such as CD69, NKG2D or CXCR4 of CD3^−^NK1.1+ cells were highly expressed in these inflamed eyes, but the inhibitory receptor NKG2A was not (Fig. 1e). Interestingly, CD83 was also expressed at high levels on the CD3^−^NK1.1^+^ cells within inflamed eyes. The mean fluorescence intensity (MFI) of CD69, CD83, NKG2D and CXCR4 in the CD3^−^ NK1.1^+^ cells within the inflamed eyes were significantly greater than that obtained with CD3^−^ NK1.1^+^ cells stained by the isotype antibody (Fig. 1e, P = 0.0030, 0.0286, 0.0481, 0.0190, respectively). CD3^−^ NK1.1^+^ cells were not present in control eyes. Moreover, the expressions of CD69, CD83, NKG2D and CXCR4 on CD3^−^ NK1.1^+^ cells that were isolated from the spleen of EAU mice were also significantly increased as compared with that found in the spleens of naive mice (Fig. 1f, P = 0.0252, 0.0218, 0.0109, 0.0349, respectively. The gate for CD3^−^NK1.1^+^ cells was presented in Supplementary Fig. S4). Matured markers of NK cells, including CD11b and CD27, were detected by flow cytometry. The expressions of CD11b and CD27 on the infiltrated CD3^−^ NK1.1^+^ cells from the inflamed eyes were found to be significantly increased (Fig. 1e, P = 0.0083, 0.0351, respectively). Increased levels in the expression of CD11b on CD3^−^NK1.1^+^ cells were obtained from inflamed versus naive spleen cells, however no differences were observed in the expression of CD27 on CD3^−^ NK1.1^+^ cells between these two conditions (Fig. 1f). Of particular significance was the observation that the increased numbers of CD3^−^ NK1.1^+^ cells in the inflamed spleens and eyes were activated and expressed high levels of CD83.

### sCD83 treatment ameliorated symptoms of EAU and decreased the increased numbers of NK cells in EAU

The presence of high expression levels of CD83 in NK cells within the EAU model implies that these cells might be capable of releasing a soluble form of CD83 molecules (sCD83). Therefore, we assayed the expression of sCD83 in the serum of EAU mice as determined at the inflammatory and recovery stages. Serum concentrations of sCD83 in EAU mice were significantly increased as compared with that of control mice (Fig. 1g, P = 0.0024). Serum concentrations of sCD83 at the recovery stage in EAU mice were further increased as compared with that at the inflammatory (Fig. 1g, P = 0.0108). Such results indicate that sCD83 might participate in the recovery of EAU.

**Figure 2:**
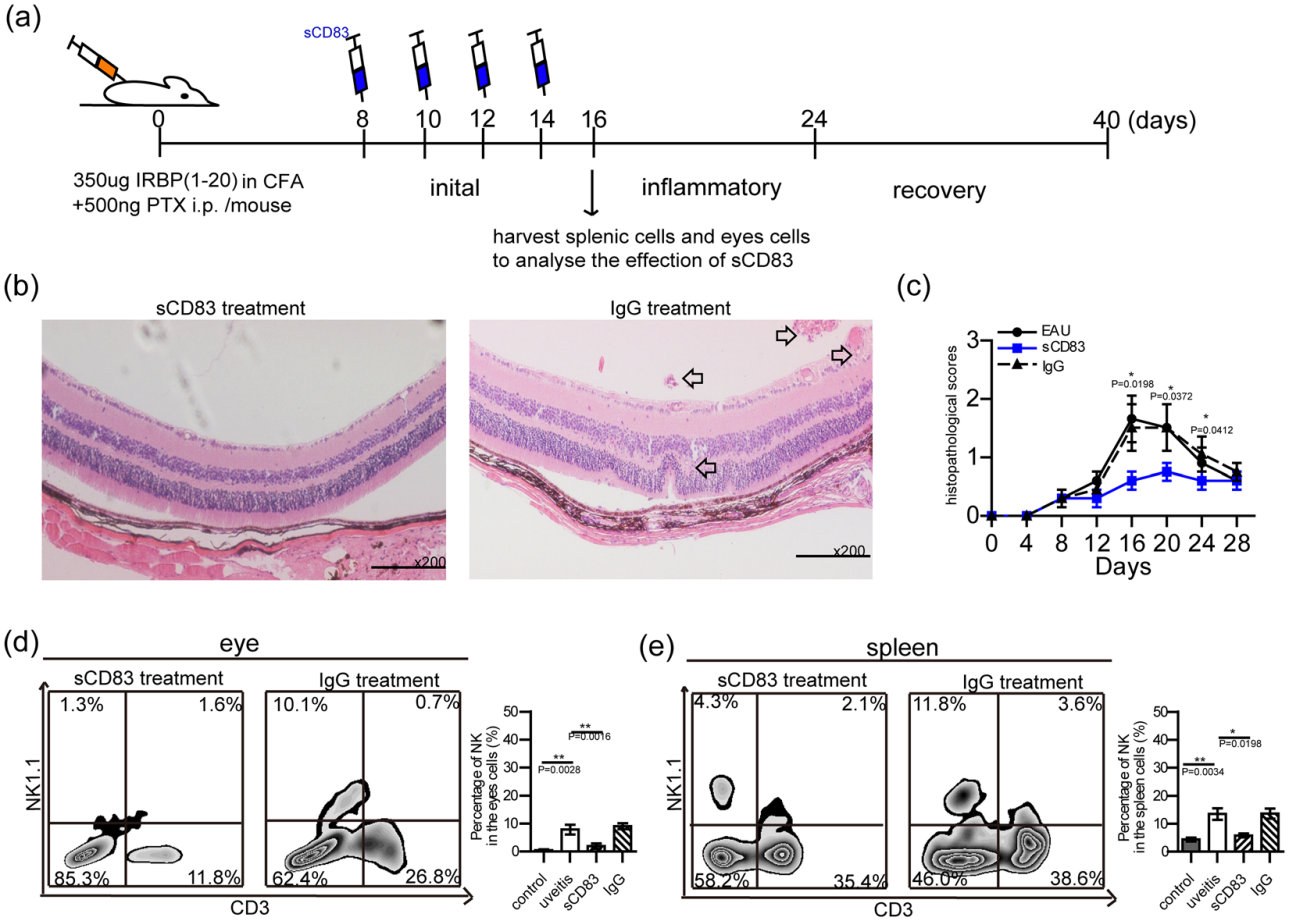
sCD83 treatment decreased pathological injury and percent of NK cells within inflamed eyes. (**a**) Mice were immunized on day 0 and treated with sCD83 on days 8, 10, 12, 14 with 10 μg sCD83 by intravenous injection. At 16 days after immunization, eyes of the mice following a treatment with sCD83 or IgG were harvested for H&E staining. (**b**) Histopathological analysis of a representative eye section from an EAU mouse treated with sCD83 or IgG. Infiltrating lymphocytes, vasculitis and photoreceptor folding within the retina were found in the eye with IgG treatment (hollow arrows). (**c**) Analysis of histopathological scores in the development of uveitis disease in EAU mice with or without sCD83 treatment or with IgG treatment. No statistically significant differences were obtained between EAU and IgG-treated-EAU mice. Statistically significant differences in histopathological scores were obtained between EAU and sCD83-treated-EAU mice on days 16, 20 and 24 (a total of twenty-four mice/group were used and the experiments were replicated three times, values represent the mean ± s.e.m, *P < 0.05). With sCD83 treatment, the percent of CD3-NK1.1+ cells from inflamed eyes (**d**) and spleen (**e**) were measured (A total of ten mice/group were used and experiments were replicated three times, values represent the mean ± s.e.m., *P < 0.05, **P < 0.01).

To further investigate the effects of sCD83 in inflammatory processes within this EAU model, these mice were intravenously injected with sCD83. In this experiment, mice were immunized on day 0, and then treated with sCD83 (10 μg/mice)^23^ on days 8, 10, 12 and 14 (Fig. 2a). The symptoms of immunized mice were assessed by histopathological examination. No obvious retinal tissue damage was observed in the inflamed eyes of immunized mice treated with sCD83 (Fig. 2b). Histological scores of inflamed eyes from immunized mice treated with sCD83 were lower than that of the eyes from mice without sCD83 treatment (Fig. 2c). However, IgG treatment did not change the retinal tissue injury and histological damage scores, compared to that seen in EAU mice (Fig. 2b,c). Clinical scores of sCD83-treated-inflamed-eyes also get the same results (Supplementary Fig. S5). Thus, sCD83 treatment exerted a protective effect in this EAU model.

In addition, the status of NK cells was also assessed in immunized mice treated with sCD83. With sCD83 treatment, the percent of infiltrating CD3^−^ NK^+^ cells within inflamed eyes significantly decreased (Fig. 2d, P = 0.0016). Moreover, the percent of CD3^−^ NK^+^ cells in splenic cells from EAU mice treated with sCD83 were also significantly decreased as compared with mice without sCD83 treatment (Fig. 2e, P = 0.0198). However, treatment of IgG did not influence the number of CD3^−^ NK^+^ cells in either the inflamed eyes or splenic cells of these mice (Fig. 2d,e). Thus, sCD83 treatment inhibited the infiltration of CD3^−^ NK^+^ cells in inflamed eyes and splenic cells. sCD83 treatment also decreased the number of infiltrated lymphocytes in these inflamed eyes as well as the percent of CD3^+^ T cells, CD3^−^B220^+^ T cells and CD11c^+^ MHC-II^+^ DCs in these eyes (Supplementary Figs S1 and S2). Moreover, upon evaluating the immune status of the spleen, we found that the percent of CD3^+^ T cells, CD11c^+^MHC-II^+^ DCs, CD11b^+^ F4/80^+^ ly6c^−^ macrophages, CD11b^+^ F4/80^−^ ly6c^+^ monocytes/neutrophils and CD11b^+^ F4/80^+^ ly6c^+^ cells neutrophil granulocytes were also all decreased (Supplementary Fig. S2). Thus, sCD83 treatment negatively regulated the immune status in the eyes and spleen within this EAU model.

As CD3^+^ T cells are believed to be a primary pathological factor in EAU, we examined the status of CD3^+^ T cells in EAU mice in response to sCD83 treatment. Our results show that sCD83 treatment decreased the expression of CD69 and Ki67 on CD3^+^ T cells in EAU, but sCD83 treatment failed to influence the expression of CD69 and Ki67 on isolated CD3^+^ T cells as assessed in *in-vitro* experiments (Supplementary Fig. S6). In this way, the capacity for sCD83 treatment to decrease the number of CD3^+^ T cells might involve effects from other immune cells. While NK cells can be a pathological factor for EAU, whether the effects of sCD83 on the immune status of EAU involve regulating NK cells requires further investigation.

### sCD83 treatments down-regulated the expression of CD11b and CD83 on NK cells in inflamed eyes and spleens

**Figure 3:**
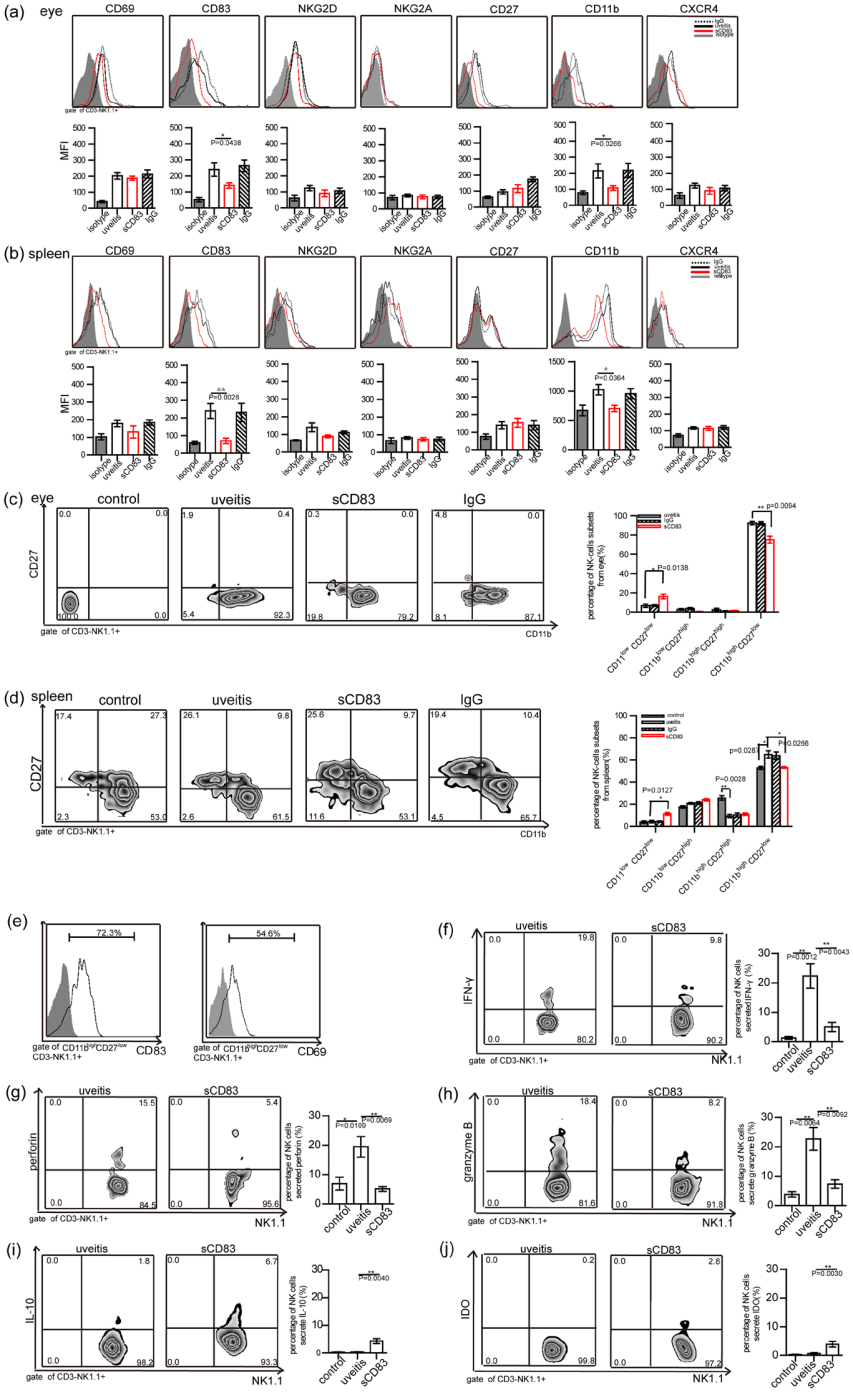
Phenotype and function of NK cells within the eyes or spleen of EAU mice treated with sCD83 as analyzed using flow cytometry. Expressions of CD69, CD83, NKG2D, NKG2A, CD11b, CD27 and CXCR4 in infiltrating CD3^−^NK1.1^+^ cells from inflamed eyes (**a**) or spleen (**b**) of EAU mice treated with sCD83 as analyzed by flow cytometry. The MFI of these molecules were analyzed and compared with NK cells obtained from inflamed eyes of EAU mice without sCD83 treatment. IgG treatment was used as a negative control. (**c**,**d**) Subsets of CD3^−^NK1.1^+^ cells infiltrating into inflamed eyes (left panel of Fig. c, a representative result from three experiments) or spleen (left panel of Fig. d, a representative result from three experiments) in EAU mice with or without sCD83 treatment. Percent of CD11b^high^CD27^low^CD83^+^CD3^−^NK1.1^+^NK-cell subsets in inflamed eyes or spleen was compared with that of sCD83 treated mice (the right bar-graph of Fig. c and d; A total of ten mice/group were used and experiments were replicated three times, mean ± s.e.m. *P < 0.05, **P < 0.01). IgG treatment was used as a negative control. (**e**) Expressions of CD69 and CD83 in CD11b^high^CD27^low^CD3^−^NK1.1^+^NK-cells were analyzed using flow cytometry. (**f**–**j**) Percent of NK cells secreting IFN-γ, perforin, granzyme B, IL-10 or IDO in response to sCD83 treatment, (a total of ten mice were used and the experiment was replicated three times, values represent the mean ± s.e.m., *P < 0.05, **P < 0.01).

To analyze the effect of sCD83 treatment on the status of NK cells in the mice subjected to inflammation, we detected the expressions of CD11b, CD27, CD69, NKG2D and CXCR4 in CD3^−^ NK^+^ cells of these mice in response to sCD83 treatment. Within the inflamed eyes, expressions of CD11b and CD83 in CD3^−^ NK^+^ cells were decreased, while expressions of CD69, CD27, NKG2D, NKG2A and CXCR4 in these CD3^−^ NK^+^ cells were not changed following sCD83 treatment (Fig. 3a). In response to sCD83 treatment, expressions of CD83 and CD11b in CD3^−^ NK^+^ cells were decreased in the inflamed spleen (Fig. 3b). These results indicate that sCD83 treatment reduced the expressions of CD11b and CD83 in NK cells.

### sCD83 treatments decreased the percent of CD11b^high^ CD27^low^CD3^−^ NK1.1^+^ NK cells in inflamed eyes and spleens

As CD11b and CD27 are important markers of NK- cell subsets, we analyzed the effect of sCD83 on NK-cell subsets in inflamed eyes and spleen. Our results revealed that 89.9 ± 2.5% of CD3^−^ NK1.1^+^ from inflamed eyes were CD11b^high^ CD27^low^ CD3^−^ NK1.1^+^ cells, 2.4 ± 1.5% of NK cells were CD11b^high^ CD27^high^ CD3^−^ NK1.1^+^ cells, 2.8 ± 0.9% of NK cells were CD11b^low^ CD27^high^ CD3^−^ NK1.1^+^ cells and 6.6 ± 1.8% of NK cells were CD11b^low^ CD27^low^ CD3^−^ NK1.1^+^ cells (Fig. 3c). With regard to the spleen, we found that the percent of CD11b^high^ CD27^low^ NK cells from the inflamed spleen was also significantly increased (64.9 ± 3.3%) as compared with that of the control spleen (52.9 ± 1.5%) (Fig. 3d, P = 0.0287). However, the percent of CD11b^high^ CD27^high^ CD3^−^ NK1.1^+^ cells from the inflamed spleen was significantly decreased (9.3 ± 1.4%) as compared with that of the normal spleen (25.6 ± 2.0%) (Fig. 3d, P = 0.0028).

With sCD83 treatment, the percent of CD11b^high^ CD27^low^ CD3^−^ NK1.1^+^ NK cells in the infiltrating NK cells of the inflamed eyes was significantly decreased (75.2 ± 3.6%) as compared with the percent of CD11b^high^ CD27^low^ CD3^−^ NK1.1^+^ NK cells in inflamed eyes without sCD83 treatment (89.9 ± 2.5%) (Fig. 3c, P = 0.0138). The percent of CD11b^high^ CD27^low^ CD3^−^ NK1.1^+^ NK cells from inflamed splenic cells was also significantly decreased (53.3 ± 0.9%) in response to sCD83 treatment as compared with inflamed splenic cells not receiving sCD83 treatment, which were increased in these inflammatory splenic cells (64.9 ± 3.3%) (Fig. 3d). With sCD83 treatment, the percent of CD11b^low^ CD27^low^ CD3^−^ NK1.1^+^ NK cells within inflamed spleen (11.2 ± 1.2%) and eyes (16.2 ± 2.4%) was significantly increased as compared with those without sCD83 treatment (4.2 ± 1.1% and 6.6 ± 1.8%, respectively) (Fig. 3c,d). Moreover, most of the activated CD11b^low^ CD27^low^ CD3^−^ NK1.1^+^ NK cells were found to express CD69 and CD83 on their surface (Fig. 3e). These data suggest that sCD83 reduced the percent of the NK1.1 cells, and CD11b^high^ CD27^low^ CD3^−^ NK1.1^+^ cells observed in the EAU model.

### sCD83 decreases the secretion of IFN-γ, granzyme B and perforin in NK cells within the EAU model

To assess whether sCD83 treatment changes the secretions of IFN-γ, perforin and granzyme B of NK cells, we examined the secretion of IFN-γ, perforin and granzyme B in NK cells of EAU mice following sCD83 treatment. Our results showed that sCD83 treatment significantly decreased the secretions of IFN-γ, perforin and granzyme B in NK cells of EAU mice compared with that without sCD83 treatment (Fig. 3f–h, P = 0.0043, 0.0069 and 0.0092, respectively). In these inflammatory mice, the secretions of IFN-γ, perforin and granzyme B in NK cells were significantly increased versus that in untreated control mice (Fig. 3f–h, P = 0.0012, 0.0109 and 0.0064, respectively). However, sCD83 treatment increased the expression of IL-10 and IDO in NK cells within EAU mice (Fig. 3i,j). These results indicate that sCD83 might influence the secretion of cytokines and cytotoxic factors of NK cells present in EAU mice.

### sCD83 decreased the expression of CD11b and CD83, and the secretion of cytotoxic factors of NK cells as determined *in vitro*

**Figure 4:**
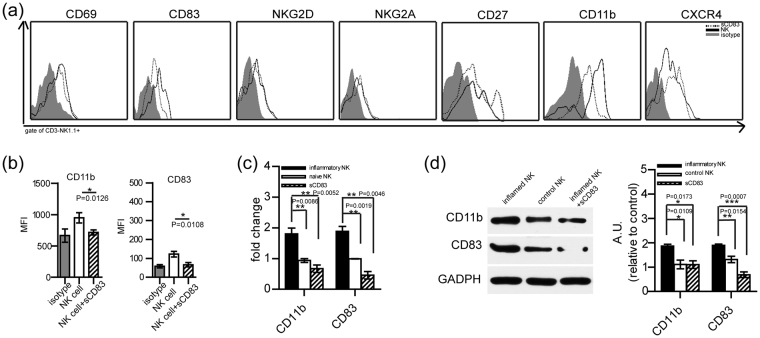
Expressions of CD11b and CD83 in NK cells following sCD83 treatment was analyzed *in vitro*. (**a**) Expressions of CD69, CD83, NKG2D, NKG2A, CD11b, CD27 and CXCR4 in NK cells with or without sCD83 treatment. (**b**) Mean fluorescence values of CD11b and CD83 in CD3^−^ NK1.1^+^ cells with or without sCD83 treatment (three separate experiments were repeated, values represent the mean ± s.e.m., CD11b: P = 0.0126, CD83: P = 0.0108). (**c**) Quantitative real-time reverse-transcriptase–PCR (qRT–PCR) of CD11b and CD83 expression in isolated NK cells from EAU mice (black bar), naive mice (open bar) and sCD83 treated isolated NK cells from EAU mice (gray bars) (mean ± s.e.m. **P < 0.01). (**d**) Western-blot analysis of CD11b, and CD83 expression in isolated NK cells from EAU mice (black bar), control mice (open bar) and sCD83 treated isolated NK cells from EAU mice (gray bars) (values represent the mean ± s.e.m. *P < 0.05, **P < 0.01, ***P < 0.001).

As an approach to examine any potential direct effects of sCD83 on NK cells, we isolated NK1.1^+^ cells from the spleen of EAU mice at 16 days post-immunization. Then 10^6^/ml of isolated NK1.1^+^ cells was treated with 10 ng/ml sCD83 for 24 h. With this protocol, we observed a reduction in the expressions of CD83 and CD11b within NK cells treated with sCD83. MFI of CD83 or CD11b was also decreased in NK cells following sCD83 treatment, as compared with NK cells not receiving sCD83 (Fig. 4a,b). However, no significant changes in the expressions of CD69, CD27, NKG2D, NKG2A, and CXCR4 in NK cells were obtained in response to sCD83 (Fig. 4a). These results provide support for the proposition that sCD83 can directly influence the expressions of CD11b and CD83 within NK cells.

To further examine the potential for direct effects, we sorted NK1.1+ cells from the spleen of EAU mice and treated these cells with sCD83 for 24 hours. We then extracted RNA from these cells to analyze the transcriptional expressions of CD11b and CD83 using QPCR. However, with sCD83 treatment, the expression of CD11b within NK cells of mice subjected to inflammation was lower than that of EAU mice without sCD83 treatment, which expressed high level of CD11b versus that of naive NK cells (Fig. 4c). Within inflamed NK cells treated with sCD83, the expression of CD83 was lower than that without sCD83 treatment (Fig. 4c). Results of western blot experiments showed that the expressions of CD11b and CD83 within inflamed NK cells were lower following sCD83 treatment versus inflamed NK cells without sCD83 treatment, which expressed higher levels of CD11b and CD83 than that observed in naive NK cells (Fig. 4d). These data provide further support for a direct effect of sCD83 treatment in decreasing the expressions of CD11b and CD83.

**Figure 5:**
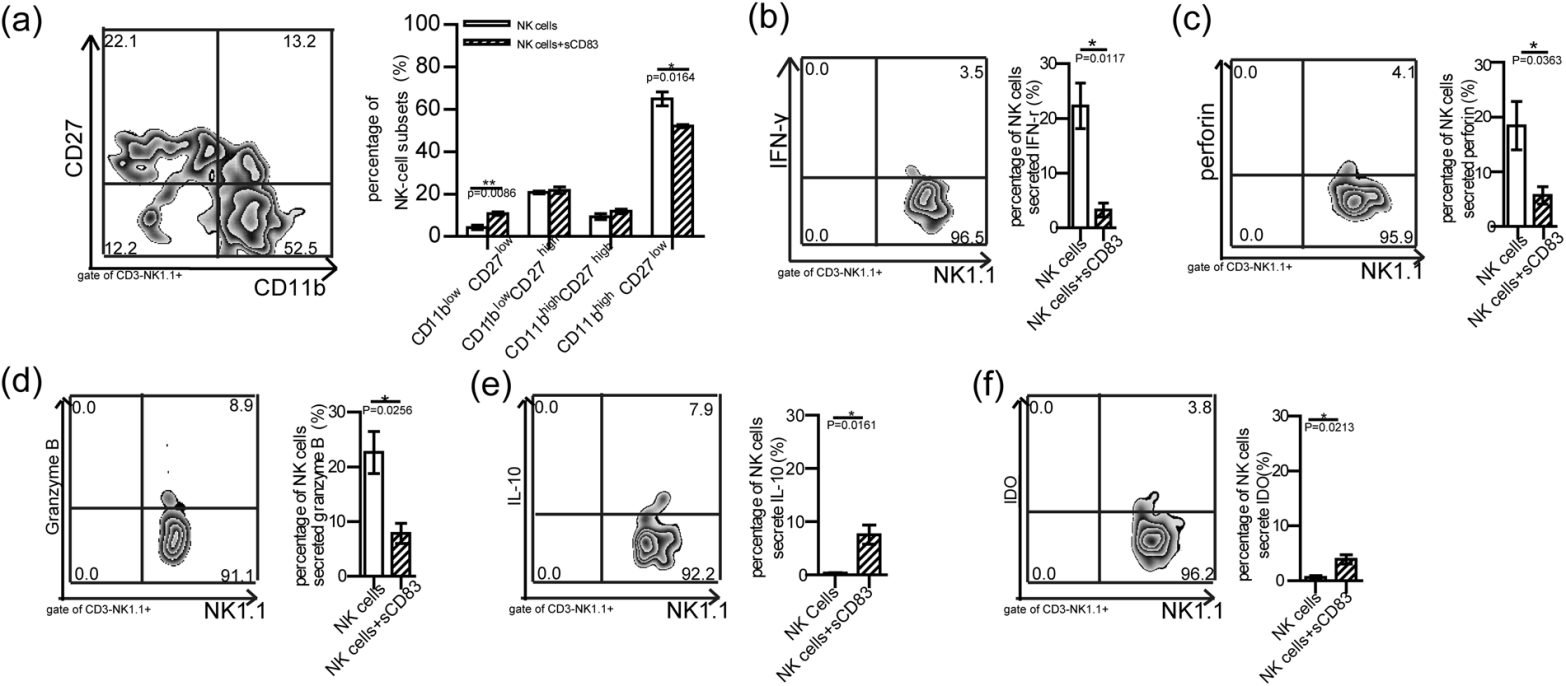
Mature subsets and functions of NK cells with sCD83 treatment were analyzed *in-vitro*. (**a**) Percent of individual NK cell-subsets in NK cells which were isolated from EAU mice with or without sCD83 treatment. (**b-f**). Percent of NK cells secreting IFN-γ (**b**), perforin (**c**), granzyme B (**d**), IL-10 (**e**) and IDO (**f**) following sCD83 treatment as assayed using flow cytometry (the experiments were replicated three times, values represent the mean ± s.e.m., *P < 0.05, **P < 0.01).

As sCD83 decreased the expression of CD11b in NK cells, we next examined the proportion of NK cell subsets that might be responsive to sCD83. Treatment with sCD83 produced a significant decrease in the percent of CD11b^high^ CD27^low^ CD3^−^ NK1.1^+^ NK cells in NK cells (Fig. 5a, P = 0.0164). However, the percent of CD11b^low^ CD27^low^ CD3^−^ NK1.1^+^ NK was significant increased (Fig. 5a P = 0.0086). Additionally, the percent of NK cells secreting IFN-γ, perforin or granzyme B was decreased after sCD83 treatment (Fig. 5b–d). However, with sCD83 treatment, the expression of IL-10 and IDO in NK cells was increased (Fig. 5e,f).

### Transferring sCD83-treated NK cells into EAU mice to ameliorate the symptoms and decrease the increasing numbers of lymphocytes in EAU

**Figure 6:**
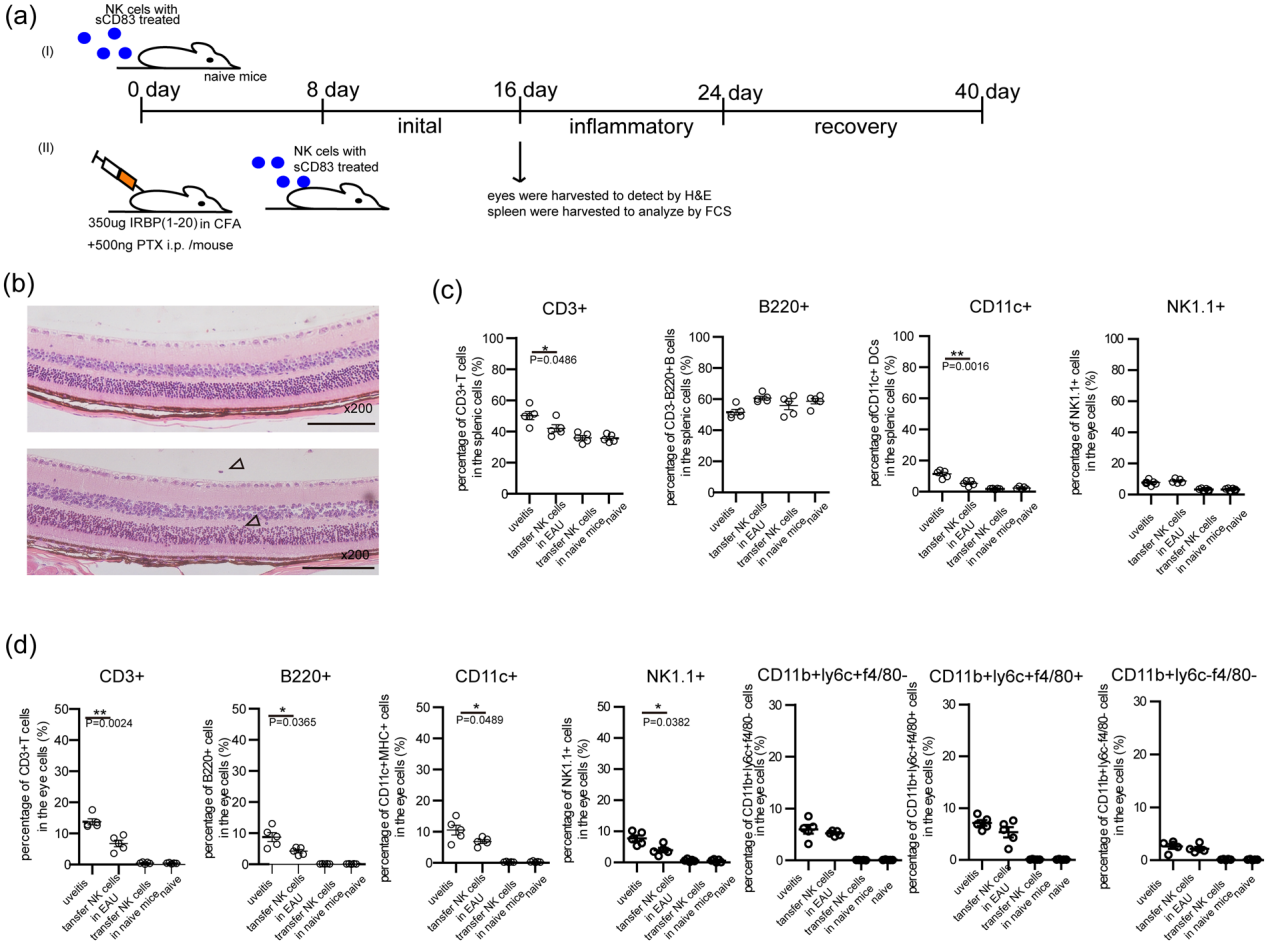
Reductions in symptoms and the number of lymphocytes in EAU mice by sCD83-treated-NK cells. (**a**) C57BL/6 Mice were immunized on day 0 and sCD83 treated-NK cells were transferred into mice by intravenous injection. At 16 days after immunization, eyes of the mice receiving or not a transfer of NK cells were harvested for H&E staining. Eyes and spleen were also harvested from untreated naïve mice that received a transferred by sCD83 treated-NK cells for H&E staining. (**b**) Pathological changes of inflamed eyes from EAU and naive mice receiving a transfer of sCD83 treated-NK cell. Some infiltration of lymphocytes and slight photoreceptor folding were observed in the eyes of EAU mice receiving sCD83 treated-NK cells (hollow arrows). (**c**) Subsets of lymphocytes from the spleen of EAU and naive mice receiving or not sCD83 treated-NK cells. (**d**) Subsets of lymphocytes in the eyes of EAU and naïve mice receiving or not sCD83 treated-NK cells. (A total of five mice/group were used for three separate experiment, values represent the mean ± s.e.m., *P < 0.05, **P < 0.01).

In continuing with this investigation, a final series of experiments were performed as below: the 10^7^/ml NK cells were obtained from EAU mice, which were immunized after 16 days, and then treated with 10ng/ml sCD83 for 24 h. These cells were then transferred into naïve (Fig. 6a (i)) or EAU (Fig. 6a (ii)) mice. At 16 days after transfer, no obvious retinal tissue damage was found in the inflamed eyes of these naive mice (Fig. 6b, upper panel), nor did this treatment influence the subsets of lymphocytes in these naïve mice (Fig. 6c,d). When sCD83-treated NK cells were transferred into the EAU model, the symptoms within these mice were assessed by histopathological examination at 8 days after sCD83-treated NK cells transferred (Fig. 6a (ii)). Small amounts of infiltrated lymphocytes were found and the outer nuclear layer of retinal tissue was slightly deformed in these mice (Fig. 6b, bottom panel, hollow arrows). The histopathological scores and clinical scores of the naïve mice with sCD83-treated NK cells transfer were lower than that of EAU mice; and the histopathological scores and clinical scores of the EAU mice with sCD83-treated NK cells transfer were also lower than that of EAU mice (Supplementary Fig. 7 a and b). Moreover, this transfer of sCD83-treated NK cells significantly decreased the percent of CD3^+^ T and CD11c^+^ DCs cells in the spleen of this EAU model (Fig. 6c, P = 0.0486 and 0.0016, respectively) and the percent of CD3^+^ T cells, B220^+^ B cells and CD11c^+^ DCs, CD3^−^ NK1.1^+^ NK cells in inflamed eyes (Fig. 6d, P = 0.024, 0.0365, 0.0489 and 0.0382, respectively). However, no effects of this treatment were observed upon the percent of CD11b^+^ F4/80^+^ ly6c^−^ macrophages, CD11b^+^ F4/80^−^ ly6c^+^ monocytes/neutrophils and CD11b^+^ F4/80^+^ ly6c^+^ cells neutrophil granulocytes in the spleen (data not shown) and within eyes (Fig. 6d).

## Discussion

A number of studies have reported that NK cells produce an increase in autoimmune uveitis^5,9,31,33^, but the exact role of these NK cells in autoimmune uveitis remains controversial. Differences in the roles played by NK cells in EAU might be related to variations in NK cell status that exist at different stages of the disease. For example, activated NK cells which produced IFN-γ, were correlated with the pathogenesis of autoimmune uveitis^5,33^, while those failing to produce IFN-γ, were correlated with remission of autoimmune uveitis^5^. Our data showed that the number of CD3^−^ NK.1.1^+^ cells were increased in inflamed eyes and spleens at the inflammatory phase and these cells expressed high levels of CD69 and NKG2D, but not NKG2A, on their surface. Moreover, these NK cells secreted increased amounts of IFN-γ, perforin or granzyme B. We also demonstrated that transferring CD3^−^NK.1.1^+^ cells extracted from the EAU model to naive mice induced damage within ocular tissue. Collectively, these findings indicate that the increases in CD3^−^NK.1.1^+^ cell numbers might play a pathogenic role in EAU at the inflammatory phase.

**Figure 7:**
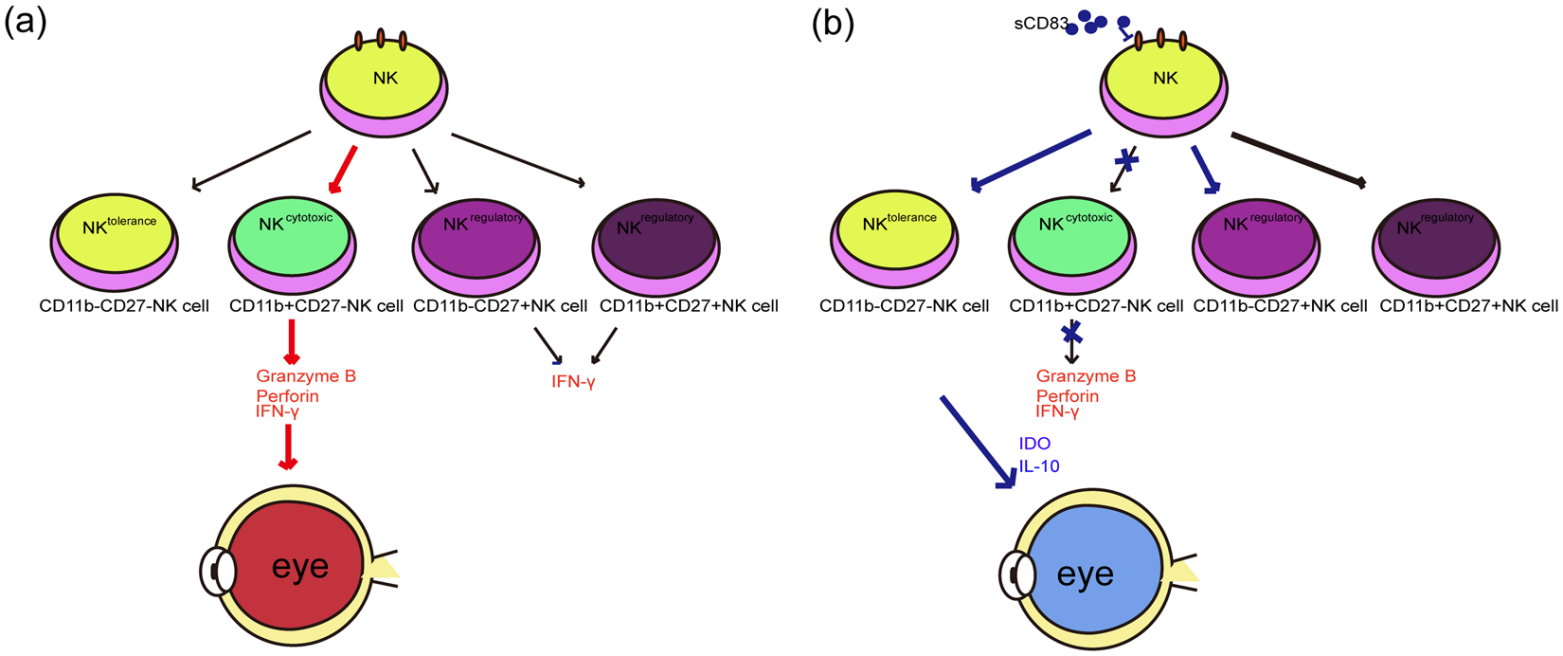
Diagram of the role of NK cells in the EAU model and effects of sCD83 regulation of the NK cells involved with ameliorating effects of EAU. (**a**) NK cells in EAU consist primarily of CD11b^high^ CD27^low^ NK cells, which secrete Granzyme B, perforin and IFN-γ to result in pathological ocular damage. (**b**) sCD83 treatment decreases the expression of CD11b on NK cells to decrease the proportion of CD11b^high^ CD27^low^ NK cells, thereby increasing the proportion of CD11b^low^ CD27^low^ NK cells in the periphery to reduce the cytotoxic effect of NK cells.

Although subsets of NK cells can function as an important source of lymphocytes with cytotoxic activity, and have been reported to be increased in Behcet’s disease^5,33^, there are no studies in which NK-cell subsets have been shown to be pathogenic in the EAU model. Our data demonstrate that CD11b^high^ CD27^low^ CD3^−^ NK.1.1^+^ cells represent a major subset of increased NK cells present in inflamed spleens and eyes, and these cells expressed high levels of CD83 and CD69 on their cell surface. Moreover, the increased amounts of granzyme B, perforin, and IFN-γ secretion in these NK cells within EAU mice provides further evidence indicating a cytolytic role resulting from elevated numbers of NK cells in EAU. CD11b^high^ CD27^low^ NK cells exhibit high cytolytic function and are referred to as NK^cytotoxic^ ^16,17^. Thus, the CD11b^high^ CD27^low^CD3^−^NK.1.1^+^ cells which are increased and secrete granzyme B, perforin, and IFN-γ in the EAU model appear to be a critical factor responsible for the damage present in this model (Fig. 7a). Specifically, it seems likely that the CD11b^high^ CD27^low^ CD3^−^NK.1.1^+^ cells present in EAU might infiltrate the eyes and play a cytolytic role resulting in damage to retinal tissue.

sCD83 displays a strong immune-suppressive role in inducing the tolerance of DCs, inhibiting monocyte differentiation into dendritic cells and inhibiting T-cell activation by regulating the status of DCs and monocytes^20,21,28,37^. An examination of the crystal structure of sCD83 has revealed that its immune-suppressive activities might result from interactions with the proteins that present in immunological synapses^38^. However, the effects of sCD83 in immune responses remain unclear and there are no reports on the effects of sCD83 in NK cells. Our current results show that sCD83 affects the status of NK cells in EAU. In specific, we report here that sCD83 treatment decreased the expressions of CD11b and CD83 and the secretions of granzyme B, perforin and IFN-γ in NK cells. In addition, we found that transferring sCD83 treated-NK cells into the EAU mouse model reduced the amount of retinal tissue damage. Taken together, these findings demonstrate that sCD83 negatively regulated the status of NK cells.

One possible mechanism for this effect of sCD83 on NK cells might be through a reduction in the expression of CD11b in NK cells. CD11b represents an important marker for mature NK cells and might affect the status of NK cells via three potential processes: 1) CD11b forms the integrin alpha-M beta-2 molecule (Mac-1) by combining with CD18, and is associated with migration and adhesion of innate immune cells^39^. In our study, sCD83 did decrease the expression of CD11b, while did not influence the expression of CXCR4 in NK cells. Thus, sCD83 might affect NK cell infiltration into the eyes through a vascular adhesion or lymphatic infiltration pathway by decreasing the expression of CD11b in NK cells, 2) CD11b is a marker for division of NK cell subsets^16^. Thus, sCD83 treatment could decrease the percent of CD11b positive subsets by decreasing the expression of CD11b in NK cells. In our experiments, sCD83 treatment decreased the percent of CD11b^high^ CD27^low^ NK cells, a cytotoxic subset of NK cells^16^, both *in vivo* and *in vitro*. Thus, sCD83 may ameliorate the development of EAU by decreasing the percent of the CD11b^high^ CD27^low^ NK subset thereby reducing the cytotoxic effects of NK cells upon retinal tissue. Moreover, sCD83 treatment increased the percent of the tolerance subset, CD11b^low^ CD27^low^ NK cell, which might promote the recovery of immune balance in ocular tissue. Although CD11b^low^ CD27^low^ NK cells are tolerance subsets, they still express the activation markers CD69 and killer cell immunoglobulin-like receptors^40^, which might provide an explanation of why the expressions of CD69, NKG2D, NKG2A in NK cells were not influenced by sCD83. and 3) CD11b is a marker for mature NK cells and participates in the function of NK-cell subsets^16,41^. Altering the maturational status of NK cells has critical consequences for NK cell function^42^. Thus, sCD83 clearly regulates NK cell function, most notably by decreasing the mature subsets of NK cells and regulating the function of NK cells through decreasing the expressions of CD11b and CD83 in NK cells. Such effects may then serve as the basis for the ameliorative effects of sCD83 in the EAU model (Fig. 7b). Additionally, sCD83 decreased the expression of mCD83 on NK cells at transcriptional and protein levels. It is possible that sCD83 may either be conveying a negative signal through binding with its ligand or lacking a positive signal that might influence the expression of mCD83 on NK cells. However, this hypothesis remains to be tested. sCD83 was reported to affect the maturation of DCs and monocytes^20,22,24^, which could modulate the function and migration of NK cells in multiple synergistic feedback loops driven by cell–cell contacts and the secretion of cytokines^43–47^. Thus, it is possible that sCD83 could influence the function of NK cells by regulating the status of other immune cells. A complete understanding of the exact mechanisms of these effects of sCD83 upon NK cells will require further investigation.

Immunotherapy is the main treatment protocol for autoimmune uveitis, but it is clear that further studies are needed to identify the most effective target^30^. In this study, we showed that sCD83 might serve as a potential therapeutic strategy for autoimmune uveitis. In EAU mice model, sCD83 ameliorated the degree of ocular tissue damage and reduced the number of infiltrated lymphocytes into the eyes (Fig. 2 and Supplementary Figure S2). Ocular inflammatory cell infiltration and systemic immune disorders represent the salient characteristics of uveitis^29,30,34^. sCD83 not only changed the number of ocular infiltrating lymphocytes, but also altered the number of lymphocytes within the spleen suggesting that sCD83 influences inflammatory cells associated with the eyes as well as the systemic immune system. It is possible that sCD83 might affect the lymphocytes in the eyes by direct flow into eyes through aqueous humor and/or by suppressing peripheral lymphocytes from infiltrating the eyes. Additionally, as sCD83 negatively regulates the function of NK cells, it also has the potential to ameliorate the symptoms of EAU by affecting the status of T cells or DCs. The exact effects of sCD83 on lymphocytes in EAU will require additional investigation.

Based upon our current results, we propose that sCD83 has a potential role in treating autoimmune uveitis. The beneficial mechanisms of sCD83 in ameliorating ocular tissue damage, in part, involve a reduction in the expression of CD11b in NK cells, which then decreases the percent of CD11b^high^ CD27^low^ CD83^+^ CD3^−^ NK1.1^+^ cells, a mature NK-cell subset (Fig. 7). Further studies are warranted to deduce any additional roles that sCD83 may play in the treatment of autoimmune uveitis and its regulatory mechanism in NK cells.

## Materials and Methods

### Experimental autoimmune uveitis (EAU)

Pathogen-free female C57BL/6 (B6) (6–8 weeks) mice were purchased from Peking Vital River Laboratory Animal Ltd., Beijing, China and were maintained in specific pathogen-free conditions according to the guidelines for the care and use of laboratory animals as published by the China National Institute of Health. All animal procedures were approved by the ethical committee of Shandong Academy of Medical Sciences (Jinan, China). The induction of EAU in C57/B6L mice has been described previously^35,36^. Briefly, C57BL/6 mice were immunized subcutaneously at 6 locations (on the footpads, tail base, the back of neck and two flanks) with 350 μg human interphotoreceptor retinoid-binding protein peptide (IRBP)_1–20_ (ChinaPeptides Co., Ltd., Shanghai, China) that was emulsified in complete Freund’s adjuvant (Sigma, St. Louis, MO, USA). Subsequently, a single dose of 500 ng pertussis toxin (PTX, Enzo Life Sciences, Farmingdale, YN, USA) was injected intraperitoneally. After immunization, the mice were examined by histopathological examination on days 0, 4, 8, 12, 16, 20, 24, 28, 32, and 36. The disease was graded using scoring systems as previously described^36^. Clinical scoring was tested by using a Genesis-D camera (Kowa Company Ltd., Hamamatsn City, Japan) as previously described^36^. The concentration of sCD83 in the serum and aqueous humor was analyzed with use of an ELISA kit of mouse sCD83 (Uscn Life science Inc, Wuhan, Hubei, China).

### sCD83 treatment

sCD83 protein is coded by a DNA sequence encoding the extracellular domain of mouse CD83 (O88324) (Met 22-Arg 133) and is fused with the Fc region of human IgG1 at the C-terminus. It was synthesized and purchased from Sino Biological Inc., Beijing, China. On day 8 after immunization, the mice were treated with sCD83 (10 μg/mouse) through an intravenous injection. sCD83 was injected on alternate days. The severity of retinal tissue damage was analyzed with use of H&E staining. Human IgG1 (10 μg/mouse), administered through an intravenous injection, was used as the control.

### Histopathological examination

The local inflammation within the eye was confirmed by histopathological examination. Eyes were harvested from immunized mice on days 0, 4, 8, 12, 16, 20, 24, 28, 32, and 36, and were fixed for 48 h in FAS fixing solution (Wuhan Goodbio technology CO., Ltd, Wuhan, Hubei, China). The fixed tissues were embedded in paraffin, sectioned (4–6 μm) through the papillary–optic nerve plane, and stained with hematoxylin and eosin (H&E). They were observed under a microscope (ECLIPSE Ti-s, Nikon, Japan) and the disease was graded on the basis of cellular infiltration and structural changes.

### Isolation of cells from inflamed eyes or spleens

The eyes were collected from the mice as reported previously^31^. Briefly, the lens and the cornea of eyes were removed. A single cell suspension was prepared by digestion for 10 min at 37 °C with collagenase (1 mg/ml) and DNAse (100 ug/ml) in RPMI-1640. The eye-infiltrating cells were obtained using this protocol, which consisted of inflammation-recruited immune cells.

Spleen cells were obtained from naive and EAU mice after immunization. The cells were collected by Ficoll-Hypaque density gradient centrifugation and cultured at 37 °C in a 5% CO_2_ incubator for flow cytometry analysis.

### Isolation of NK cells

NK cells from spleen were isolated using anti-mouse NK1.1-PE antibody (eBioscience, San Diego, CA, USA) and anti-PE MicroBeads (Miltenyi Biotec, Bergisch Gladbach, Germany), according to the manufacturer’s instructions. These NK cells were cultured in the RPMI 1640 (Sigma, St. Louis, MO, USA) containing 10% FBS (Atlanta Biologicals, Atlanta, GA, USA) and supplemented with 1 mM L-glutamine, 50 µM 2-mercaptoethanol (2-ME), 1 mM non-essential amino acids and 40 ng/ml recombinant murine IL-2 (R&D Systems, Minneapolis, MN, USA) at 37 °C in a 5% CO_2_ incubator.

### Flow cytometric analysis

Aliquots of 1 × 10^6^ cells were stained with different monoclonal antibodies, according to the protocol for corresponding antibodies. After being incubated for 30 min and washed twice, cells from each sample were analyzed using FACSVerse and the CellQuest data acquisition and analysis software (BD Biosciences, USA). To assess intracellular cytokine expression, we stimulated the prepared cells for 5 h with cell stimulation cocktail (eBioscience, San Diego, CA, USA) at 37 °C in a 5% CO_2_ environment. The cells were then harvested and transferred to tubes, washed once with PBS, and incubated with fluorescent antibodies of CD3ε, NK1.1, CD27, CD11b, CD69, CXCR4, NKG2D, NKG2A, IFN-γ, perforin, and granzymes B conjugated with corresponding fluorescent dyes (eBioscience, San Diego, CA, USA) according to the manufacturer’s instructions.

### Real-time RT-PCR

Total RNA of isolated NK cells was extracted using TRIzol Reagent (Invitrogen, Carlsbad CA, USA) and cDNA was synthetized using the RevertAid First Strand cDNA Synthesis kit (Thermo Scientific, Rockford, IL,USA) according to the manufacturers’ instructions. Real-time PCR was performed with specific primers for CD11b (forward, 5′-CTTTGGGAACCTCCGACCAG-3′ and reverse, 5′-CACCAAAGTGCCAAGCCCA-3′) CD83 (forward, 5′-GGCCTATTCCCTGACGATCC-3′ and reverse, 5′-TTGGGGCATCCTTCAGAACC-3′). Relative gene expression was calculated using the 2^−ΔΔT^ method. Data were normalized by the housekeeping gene (β-actin) and expressed as fold change relative to the control.

### Western blot

Isolated NK cells were lysed with RIPA buffer (Beyotime Biotechnology, Shanghai, China). Identical quantities of protein were separated by 10% SDS-PAGE and transferred to polyvinylidend difluoride (PVDF) membranes. Subsequently, 5% non-fat dry milk in Tris-buffered saline 0.1% Tween 20 (TBS-T) was used to block non-specific binding sites for 1 h. After washing with TBS-T, membranes were incubated with primary antibodies against mouse CD11b (Abcam, Cambridge, MA, USA), anti-CD83 (Abcam, Cambridge, MA, USA), β-actin (cell signaling Technology, Beverly, MA, United States) at 4 °C for overnight. Membranes were then washed and incubated with secondary antibodies goat-anti-rabbit IgG antibodies conjugated to HRP (Beyotime Biotechnology, Shanghai, China) for 1 h. Finally, the membranes were developed using the Super Signal West pico Chemiluminescent Substrate (Thermo Scientific, Rockford, IL). Densitometric analyses were performed using the ImageJ software (NIH, Bethesda, MD, USA).

### Statistical analysis

Data analysis was performed using GraphPad Prism 5 (GraphPad Software, San Diego, CA). Each experiment was performed in duplicate and replicated three times. Two-tailed Student’s t-tests were applied to determine the statistical significance between two groups. ANOVA tests were applied for the multiple sets of data. The scores of uveitis pathology were analyzed by Mann–Whitney U tests for the two independent samples of this nonparametric test, or Kruskal-Wallis test for the multiple independent samples of this nonparametric test. Data were represented as mean ± s.e.m. *P* < 0.05 (*), 0.01 (**) and 0.001(***) were required for results to be considered statistically significant.

## Electronic supplementary material

Supplementary Figure and Figure legends
