# Supplementary material for: NK cells are negatively regulated by sCD83 in experimental autoimmune uveitis
Source: Sci Rep. 2017 Oct 16;7:12895. doi: 10.1038/s41598-017-13412-1 (PMC5643513; doi:10.1038/s41598-017-13412-1)
Supplement: Supplementary file 1 — Supplementary Figure and Figure legends [file 41598_2017_13412_MOESM1_ESM.pdf]

NK cells are negatively regulated by sCD83 in experimental autoimmune uveitis

Wei lin1 #, Xuejing Man2*, Peng li1, Nannan Song1, Yingying Yue1, Bingqing li1, Yuanbin Li2, Yufei Sun3, Qiang Fu3#*

1 Department of microbiology, Institute of Basic medicine, Shandong Academy of medical Sciences, Jinan, 250032, China

2 Department of Ophthalmology, Yuhuangding Hospital, Yantai, China 264001

3 Department of Immunology, Binzhou Medical University, Yantai, China 264003

**co-first author, #co-corresponding author, Email: Wei Lin: weilin11@fudan.edu.cn or Linw1978@163.com; Qiang Fu:qiangfu11@fudan.edu.cn*

Supplementary Figure legends

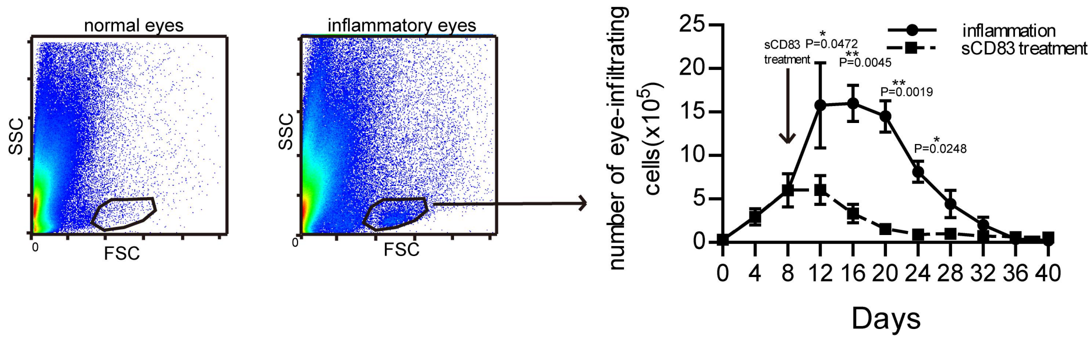

Supplementary Figure. S1. Infiltrating cells were collected in the eyes. Eyes were collected at 12-16 days post-immunization and eye-infiltrating cells were analyzed by flow cytometry. The left picture showed the infiltrated lymphocytes in the normal eyes. The middle picture showed the infiltrated lymphocytes in inflammatory eyes. The right picture showed the number of infiltrated lymphocytes in the process of EAU development (the solid line), and the number of infiltrated lymphocytes in the process of EAU development with sCD83 treatment (the dotted line). Three mice were used at every time point, and three separate experiments were repeated, values represent the mean±s.e.m., **P*<0.05,

**P<0.01.

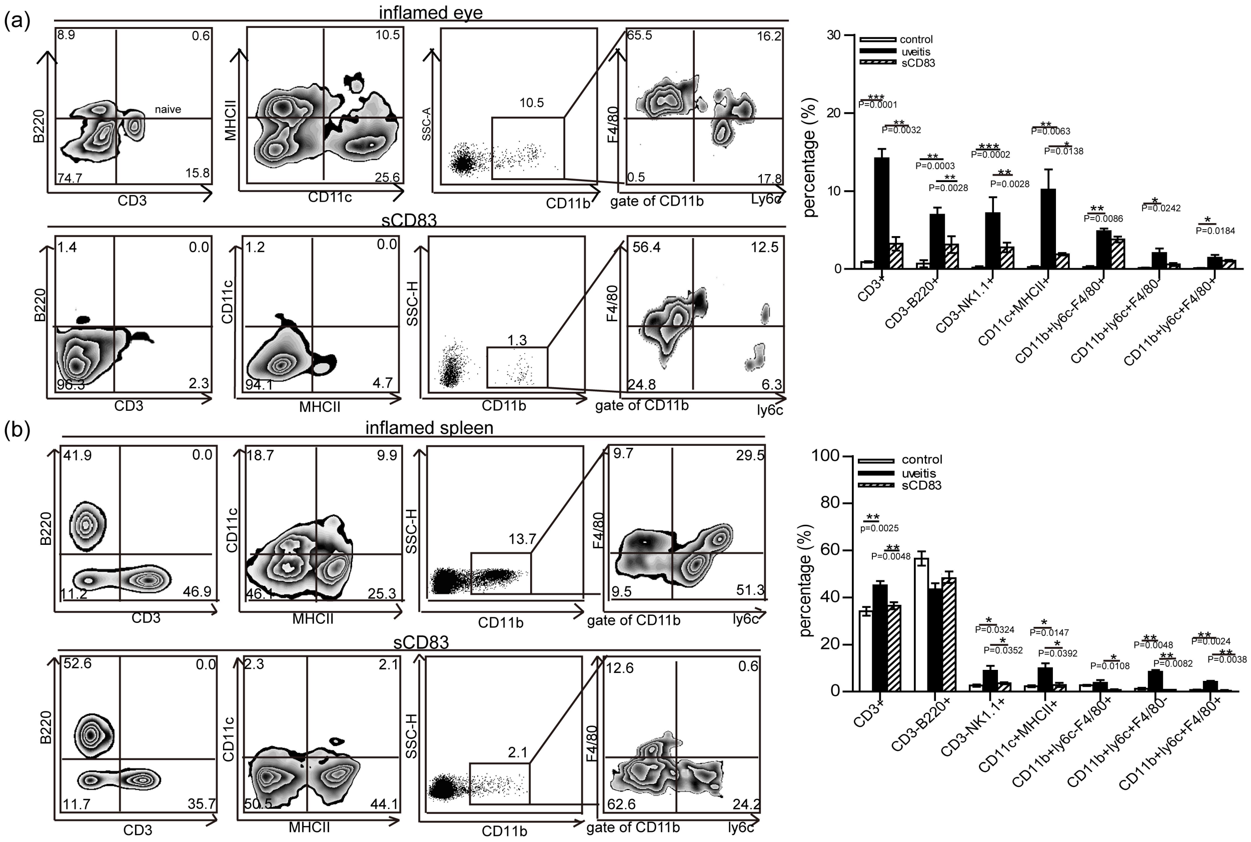

Supplementary Figure. S2. With sCD83 treatment, the percentage of CD3^{+} T cells, CD3^{-} B220^{+} B cells, CD11c^{+} MHC-II^{+} DCs, CD11b^{+} ly6c^{+} F4/80^{-} monocytes/neutophils, CD11b^{+} ly6c^{-} F4/80^{+} marcphages, and CD11b^{+} ly6c^{+} F4/80^{+} neutophils from the inflamed eyes (a) and inflamed spleen (b) were analyzed compared to those from the inflamed spleen and inflamed eyes without sCD83 treatment (a total of ten mice were used and the experiment was replicated three times, values represent the mean±s.e.m., *P<0.05,** P<0.01, ***P<0.001).

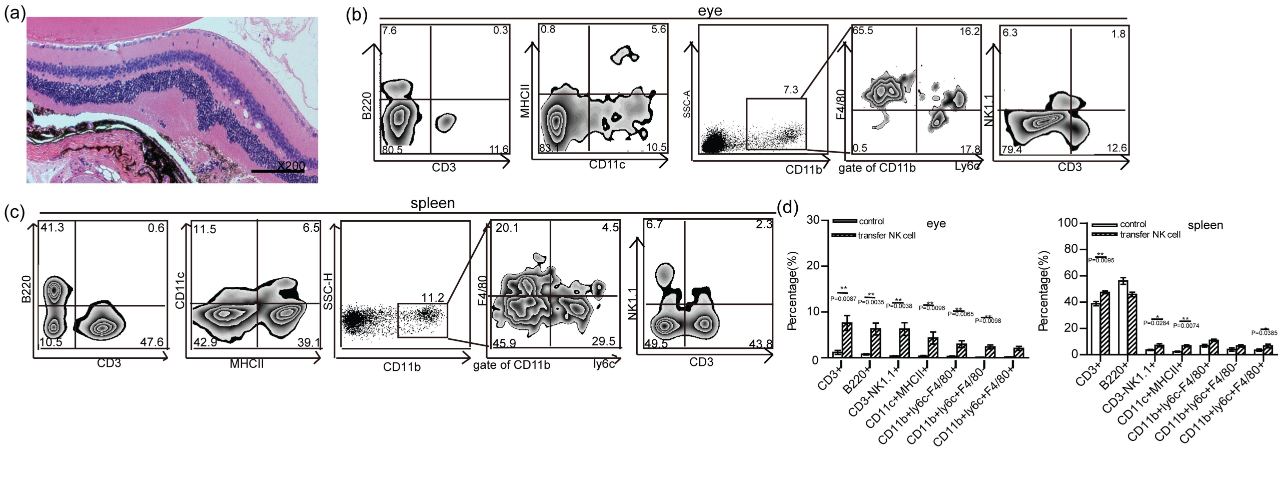

Supplementary Figure. S3. Histopathological changes within the eyes and changes in lymphocytes within naive mice following NK cell transfer. Histopathological changes within the eyes (a), lymphocyte subsets within the eyes (b) and spleen (c) of a representative naive mouse with NK cell transfer. (d) Percent of lymphocyte subsets from the eyes (left) and spleen (right) of a naive mouse with NK cell transfer as compared with that from inflamed eyes and spleen of an EAU mouse. (A total of ten mice/group were used and experiments were replicated three times, values represent the mean±s.e.m. **P*<0.05, ** *P*<0.01).

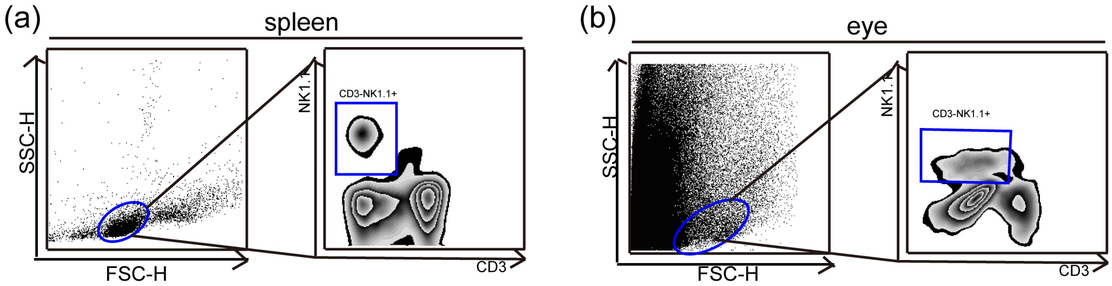

Supplementary Figure. S4. Staining controls for setting of the gates are shown. (a) Lymphocytes from spleen cells were gated (left), and then $CD3^-$ $NK1.1^+$ cells were further gated for analysis (right). (b) Lymphocytes from cells of the entire eye were gated (left), and then $CD3^-$ $NK1.1^+$ cells were further gated for analysis (right).

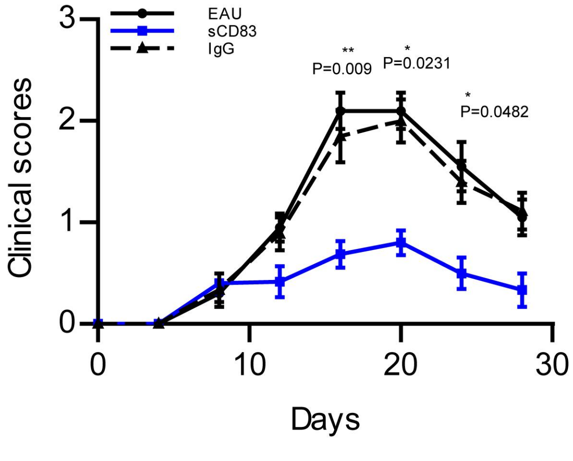

Supplementary Figure. S5. Clinical scores in the development of uveitis disease in EAU mice, sCD83-treated-EAU mice and IgG-treated EAU mice (a total of twenty-four mice/group were used and the experiments were replicated three times, values represent the mean±s.e.m, ***P*<0.01, **P*<0.05).

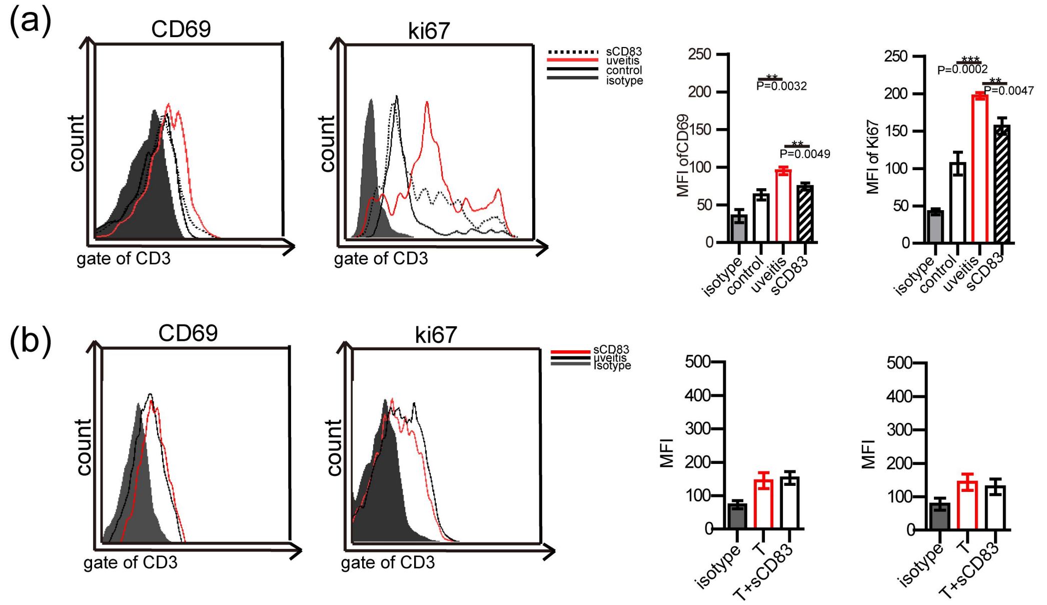

Supplementary Figure. S6. The effect of sCD83 on CD3+ T cells. (a) The expression of CD69 and Ki67 on the CD3+ T cells from control mice, EAU mice or sCD83-treated-EAU mice. (A total of ten mice/group were used and the experiments were replicated three times, values represent the mean±s.e.m. **P*<0.05, ** *P*<0.01, ****P*<0.001). (b) The expression of CD69 and Ki67 on isolated CD3+ T cells with or without sCD83 treatment. Experiments were replicated three times. Values represent the mean±s.e.m.

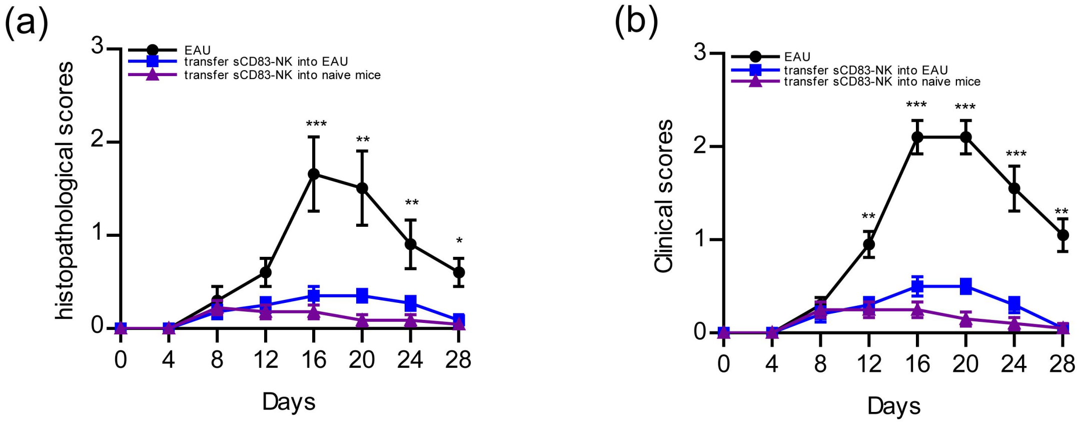

Supplementary Figure. S7. Histopathological (a) and clinical (b) scores in the development of uveitis disease in EAU mice, EAU mice with sCD83-treated-NK cell transfer and naive mice with sCD83-treated-NK cell transfer. Histopathological scores of EAU mice were significantly increased on

days 16-28 as compared with that of EAU mice receiving sCD83-treated-NK cell transfers (P values: 0.0008, 0.0026, 0.0042, 0.00247, respectively) and that of naive mice with sCD83-treated-NK cell transfers (P values: 0.0007 0.0013, 0.0035, 0.0178, respectively). Clinical scores of EAU mice were significantly increased on days 12-28 as compared with that of EAU mice receiving sCD83-treated-NK cell transfers (P values: 0.0028, 0.0005, 0.0002, 0.0009, 0.0012, respectively) and naive mice with sCD83-treated-NK cell transfers (P values: 0.0023, 0.0003, 0.0001, 0.0005, 0.0015, respectively). **(**A total of twenty-four mice were used within each group and the experiments were replicated three times, values represent the mean±s.e.m. *P<0.05, **P<0.01, ***P<0.001).
